# Sex-Specific Associations between Particulate Matter Exposure and Gene Expression in Independent Discovery and Validation Cohorts of Middle-Aged Men and Women

**DOI:** 10.1289/EHP370

**Published:** 2016-10-14

**Authors:** Karen Vrijens, Ellen Winckelmans, Maria Tsamou, Willy Baeyens, Patrick De Boever, Danyel Jennen, Theo M. de Kok, Elly Den Hond, Wouter Lefebvre, Michelle Plusquin, Hans Reynders, Greet Schoeters, Nicolas Van Larebeke, Charlotte Vanpoucke, Jos Kleinjans, Tim S. Nawrot

**Affiliations:** 1 Centre for Environmental Sciences, Hasselt University, Diepenbeek, Belgium; 2 Department of Analytical and Environmental Chemistry, Free University of Brussels, Brussels, Belgium; 3 Environmental Risk and Health Unit, Flemish Institute for Technological Research (VITO), Mol, Belgium; 4 Department of Toxicogenomics, Maastricht University, Maastricht, Netherlands; 5 Provincial Institute for Hygiene, Antwerp, Belgium; 6 Environment, Nature and Energy Department, Flemish Government, Brussels, Belgium; 7 Department of Biomedical Sciences, University of Antwerp, Antwerp, Belgium; 8 University of Southern Denmark, Institute of Public Health, Department of Environmental Medicine, Odense, Denmark; 9 Department of Radiotherapy and Nuclear Medicine, Ghent University, Ghent, Belgium; 10 Belgian Interregional Environment Agency (IRCEL), Brussels, Belgium; 11 Department of Public Health and Primary Care, Leuven University, Leuven, Belgium

## Abstract

**Background:**

Particulate matter (PM) exposure leads to premature death, mainly due to respiratory and cardiovascular diseases.

**Objectives:**

Identification of transcriptomic biomarkers of air pollution exposure and effect in a healthy adult population.

**Methods:**

Microarray analyses were performed in 98 healthy volunteers (48 men, 50 women). The expression of eight sex-specific candidate biomarker genes (significantly associated with PM_10_ in the discovery cohort and with a reported link to air pollution-related disease) was measured with qPCR in an independent validation cohort (75 men, 94 women). Pathway analysis was performed using Gene Set Enrichment Analysis. Average daily PM_2.5_ and PM_10_ exposures over 2-years were estimated for each participant’s residential address using spatiotemporal interpolation in combination with a dispersion model.

**Results:**

Average long-term PM_10_ was 25.9 (± 5.4) and 23.7 (± 2.3) μg/m^3^ in the discovery and validation cohorts, respectively. In discovery analysis, associations between PM_10_ and the expression of individual genes differed by sex. In the validation cohort, long-term PM_10_ was associated with the expression of *DNAJB5* and *EAPP* in men and *ARHGAP4* (*p* = 0.053) in women. *AKAP6* and *LIMK1* were significantly associated with PM_10_ in women, although associations differed in direction between the discovery and validation cohorts. Expression of the eight candidate genes in the discovery cohort differentiated between validation cohort participants with high versus low PM_10_ exposure (area under the receiver operating curve = 0.92; 95% CI: 0.85, 1.00; *p* = 0.0002 in men, 0.86; 95% CI: 0.76, 0.96; *p* = 0.004 in women).

**Conclusions:**

Expression of the sex-specific candidate genes identified in the discovery population predicted PM_10_ exposure in an independent cohort of adults from the same area. Confirmation in other populations may further support this as a new approach for exposure assessment, and may contribute to the discovery of molecular mechanisms for PM-induced health effects.

**Citation:**

Vrijens K, Winckelmans E, Tsamou M, Baeyens W, De Boever P, Jennen D, de Kok TM, Den Hond E, Lefebvre W, Plusquin M, Reynders H, Schoeters G, Van Larebeke N, Vanpoucke C, Kleinjans J, Nawrot TS. 2017. Sex-specific associations between particulate matter exposure and gene expression in independent discovery and validation cohorts of middle-aged men and women. Environ Health Perspect 125:660–669; http://dx.doi.org/10.1289/EHP370

## Background

Particulate matter (PM) is a complex mixture of small particles and liquid droplets that contains a number of components, including acids, organic chemicals, metals, and soil or dust particles. PM exposure is known to increase overall mortality and morbidity, mainly due to its effect on the cardiorespiratory system (Alfaro-Moreno et al. 2007; Pope et al. 2004). Exposure to PM may disturb normal physiological pathways that maintain homeostasis and this may activate cellular processes that mediate the adverse effects of PM (Kleensang et al. 2014). Gene expression changes play an important role in the activation of pathways of toxicity and gene signatures have the potential to serve as biomarkers of exposure (van Leeuwen et al. 2008; van Breda et al. 2015) and recent reports demonstrate their potential use as biomarkers of effect (La Rocca et al. 2014; Fink et al. 2014). As it has been shown previously that transcriptomic responses to diverse environmental stimuli (i.e., chemical exposure, smoking) can be significantly different between men and women (De Coster et al. 2013; Paul and Amundson 2014), we have opted to perform a sex-specific analysis.

Several studies have suggested that elevated oxidative stress may mediate toxic effects of air pollutants (Donaldson et al. 2005; Nel et al. 2001). The systemic inflammatory response following acute inhalation exposure to PM can induce leukocytosis and monocyte release from the bone marrow (Fujii et al. 2002). Controlled exposure studies of recent diesel exhaust exposure (Pettit et al. 2012) and recent exposure to ultra-fine particles (Huang et al. 2010) have reported evidence of altered gene expression in leukocytes but, to our knowledge, associations between patterns of gene expression and long-term particulate air pollution have not been studied in general populations.

## Materials and Methods

### Study Design

**Figure 1:**
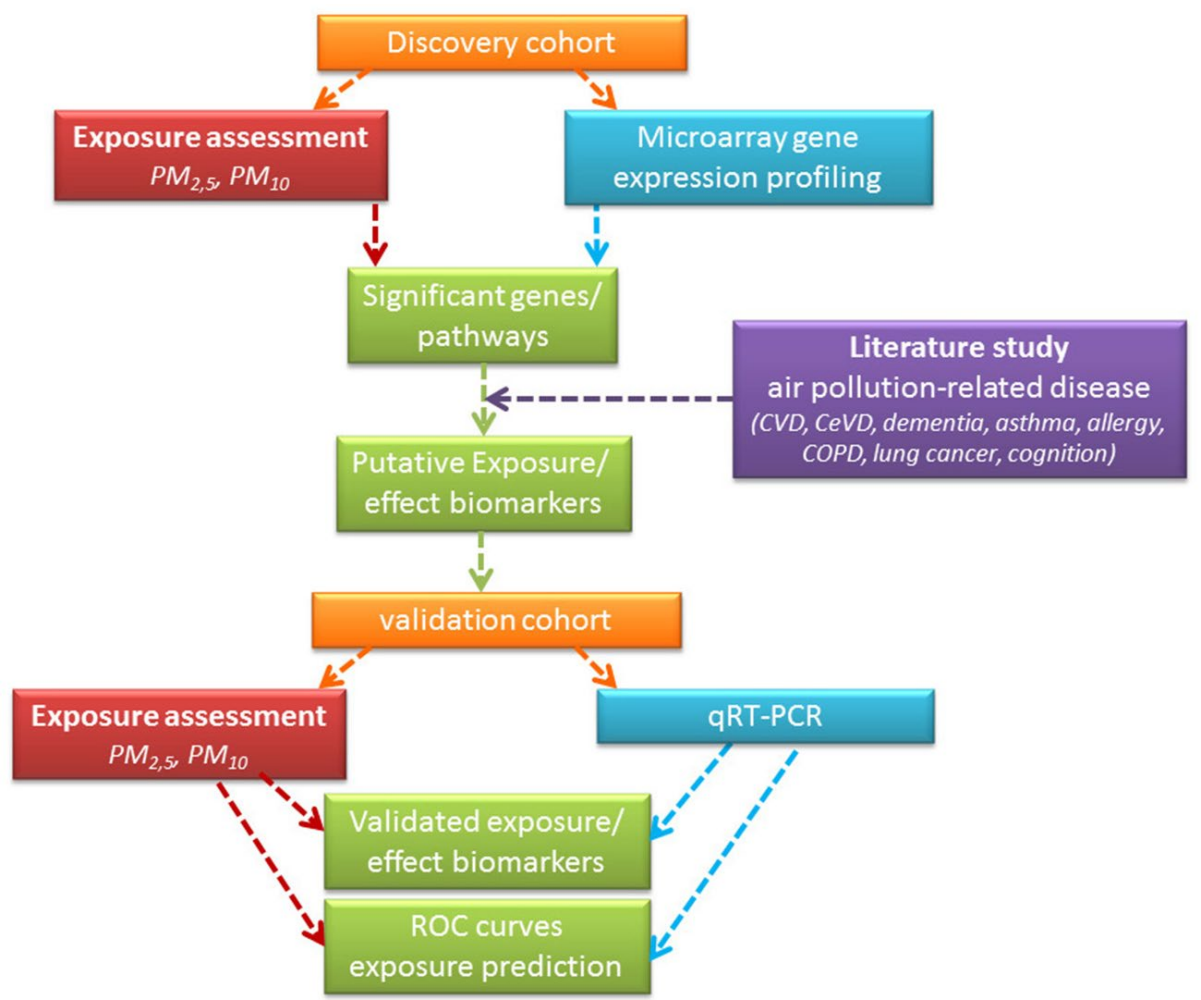
Schematic representation of the application of the modified version of the meet-in-the-middle approach to identify biomarkers of disease. Note: CeVD, cerebrovascular disease; COPD, chronic obstructive pulmonary disease; CVD, cardiovascular disease.

As our goal was to identify transcriptomic biomarkers of exposure and effect in a healthy adult population, we started by applying microarray analysis in a discovery cohort of 98 adults for which we modelled particulate matter exposure. On the resulting dataset containing significantly modulated genes and pathways, we applied a literature and bio-informatics approach to identify potential exposure effect biomarkers. Subsequently, these were validated using qPCR analysis in an independent cohort with similar characteristics as the discovery population (Figure 1). Study protocols for the discovery and validation cohort were approved by the Institutional Review Board and the Ethical Committee of Antwerp University and informed consent was obtained from all participants.

### Study Population

***Discovery cohort.*** The original study population was described previously (van Leeuwen et al. 2008) and consisted of 398 participants from eight different regions of residence in Flanders (Belgium), as part of the first Flemish Environment and Health Survey (FLEHS I) during the period 2001–2006. Participants were recruited in several communities based on random sampling. Inclusion criteria were age 50–65 years, living in Flanders > 5 years, and being able to complete questionnaires in Dutch. Prior to blood collection, informed consent was obtained from all individuals. A subset of 98 samples was selected for microarray analysis based on previously measured exposure levels to several pollutants including cadmium, lead, polychlorinated biphenyls (PCBs) (138, 153, and 180), dioxins, polycyclic aromatic hydrocarbons (PAHs), and benzene. The overall exposure to these pollutants was estimated using a *z*-score for each pollutant, and study participants with both low- and high- exposure levels were chosen for inclusion. *Z*-scores were not correlated with long-term PM_10_ exposure (*r* ^2^ = 0.0012). Smokers were excluded from the study population. PAXgene tubes (PreAnalytiX GmbH, Hombrechtikon, Switzerland) were used for RNA collection.

***Validation cohort.*** The quantitative polymerase chain reaction (qPCR) validation study was performed in an independent cohort of 175 adults who were part of the third Flemish Environment and Health Survey (FLEHS III) during the period 2012–2015. Healthy volunteers who were between 50 and 65 years of age, living at the same residential address for at least 10 years, and able to complete questionnaires in Dutch were recruited through registers of general medical practices. Prior to blood collection, informed consent was obtained from all individuals. Participants completed a questionnaire covering age, sex, and smoking habits, among other demographic characteristics; they donated blood and urine samples; and subclinical measurements including height, weight, and blood pressure were determined. The sampling campaign lasted from May 2014 until 30 December 2014. We used PAXgene tubes (PreAnalytiX GmbH, Hombrechtikon, Switzerland) to stabilize whole blood RNA for storage.

### Exposure Estimates

The PM_10_ and PM_2.5_ (10 and 2.5 μm in aerodynamic diameter) concentrations for participants’ residential addresses were calculated using a spatial temporal interpolation method (Kriging) that takes into account land cover data from satellite images [CORINE (coordination of information on the environment) land cover data set; http://www.eea.europa.eu/publications/COR0-landcover] for interpolating the measurement data of the monitoring stations from the Belgian telemetric air quality network as described previously (Maiheu et al. 2013; Jacobs et al. 2010; Janssen et al. 2008). Validation statistics of the interpolation tool gave a temporal explained variance of > 0.7 for hourly PM_10_ averages as well as for annual mean PM_10_ (Maiheu et al. 2013). In combination with the Immission Frequency Distribution Model (IFDM)] using emissions from line sources and point sources, the model chain provides daily PM_10_ and PM_2.5_ values on a 25 × 25 m receptor grid (Lefebvre et al. 2013). Our model is based on input data from 38 monitoring stations in the study area. The Initiative on Harmonisation within Atmospheric Dispersion Modelling for Regulatory Use in Europe was the incentive for intensive model intercomparison. IFDM was thoroughly compared with other models currently in use for regulatory purposes in Europe (Olesen 1995; Maes et al. 1995; Cosemans et al. 1995, 2001; Mensink and Maes 1996).

Mean daily temperatures and relative humidity for the study region were provided by the Royal Meteorological Institute (Brussels, Belgium), and apparent temperature was calculated (Steadman 1979; Kalkstein and Valimont 1986).

All our estimates were annual mean exposures over a 2-year period because we were interested in developing biomarkers for long-term exposure. For the discovery cohort, annual means were based on 2011–2012 as these were the earliest years for which detailed 25 × 25 m grid information became available. Distribution patterns were used for the year 2008. We assumed that relative differences in annual mean concentrations of particulate matter were generally consistent from year to year. For the validation cohort, annual means were based on the 2 years prior to blood sampling (i.e., 2012–2013).

### RNA Isolation

Total RNA was isolated from 2.5 mL whole blood from PAXgene Blood RNA vacutainers using the PAXgene Blood RNA system (PreAnalytiX, Qiagen, Hilden, Germany), according to the manufacturer’s instructions. A globin reduction assay (GLOBINclear™ Kit by Ambion, Austin, TX, USA) was performed in order to remove hemoglobin mRNA from samples that were submitted to microarray analysis. RNA integrity was assessed using the BioAnalyzer (Agilent, Palo Alto, CA, USA) and purity was measured spectrophotometrically. Labeled samples were checked for specific activity and dye incorporation.

### Microarray Preparation and Hybridization

We used 0.2 μg total RNA from each sample to synthesize dye-labeled cRNA (Cy3) following the Agilent one-color Quick-Amp labeling protocol (Agilent Technologies). Individual samples were hybridized on Agilent 4 × 44 K Whole Human Genome microarrays (design ID 014850).

### Microarray Data Analysis

Microarrays were scanned on an Agilent G2505C DNA Microarray Scanner (Agilent Technologies, Amstelveen, Netherlands). Raw data on pixel intensities were extracted from the scanned images using Agilent Feature Extraction Software (version 10.7.3.1; Agilent Technologies, Amstelveen, Netherlands), protocol GE1_107.sep09. Raw data were pre-processed using an in-house developed quality control pipeline in R (version 2.15.3; R Project for Statistical Computing) as follows: local background correction, flagging of bad spots, controls and spots with intensities below background, log2 transformation and quantile normalization. The R-scripts of the pipeline and additional information on the flagging can be found at https://github.com/BiGCAT-UM/arrayQC_Module. From the processed data files genes were omitted showing more than 30% flagged data, after which the data files were transferred to the Gene Expression Pattern Analysis Suite, GEPAS 2010 (Montaner et al. 2006) for further preprocessing, including merging replicate probes (based on median), and imputing missing values by means of K-nearest neighbor imputation (K = 15). Filtering for flat peaks was used with root mean square value 0.25. The filtered data, containing 28,786 genes, were used for further statistical analyses. Microarray gene expression data were analyzed and stratified for sex. In the original microarray data that set initially 28,786 unique Agilent probe IDs (out of 43,376 Agilent probe IDs) were annotated to 22,390 EntrezGene IDs. In case of multiple replicates (i.e., multiple probes for the same gene), the replicate with highest interquartile range (IQR) in relative gene expression was selected. This resulted in 15,589 unique EntrezGene IDs.

### Gene Expression Analysis

Using linear regression models adjusted for age, body mass index (BMI), socioeconomic status (SES, classified in three groups: no high school degree, high school degree, or further education degree), daytime and season of blood sampling, we obtained estimates for each gene as the log_2_-fold change in gene expression for an increment of 5 μg/m^3^ in exposure. *p*-Values < 0.05 were considered statistically significant. *p*-Values were corrected for multiplicity using the Benjamini–Hochberg false discovery rate (FDR) correction. *p*-Values corrected for multiple testing are referred to as *q*-values.

### Pathway Analysis

Gene Set Enrichment Analysis was performed utilizing the online pathway analysis tool Consensus Pathway Data Base (CPDB) (http://consensuspathdb.org/). CPDB contains ~ 5,200 pathways including protein complexes, metabolic, signaling, and gene regulatory pathways, as well as drug-target interactions. Data originate from 32 public resources curated from the literature (Kamburov et al. 2013). Gene Set Enrichment Analysis was performed in a sex-specific manner using the log_2_-fold changes of the gene expression data for all genes analyzed at the gene expression level, without preselection. For every predefined gene set in each pathway, a Wilcoxon signed-rank test was calculated, testing the null hypothesis that the distribution of their fold changes was around zero. As input, all genes without *a priori* selection (EntrezGene IDs) were uploaded with their fold changes in their gene expression. We selected the biological processes using pathways as output. The *p*-values were corrected for multiplicity and were presented as *q*-values. We defined significant biological processes and pathways by a threshold on the adjusted *p*-value (*q* < 0.05 or FDR 5%) and we included gene sets with a size between 5 and 100 members.

### Selection of Potential Exposure/Effect Biomarker Genes

We used a modified version of the meet-in-the-middle approach for biomarker identification in relation to clinical relevance (Vineis et al. 2013), a schematic representation is shown in Figure 1. We first identified the top 50 genes associated with PM_10_ (i.e., with the smallest uncorrected *p*-values) in men and women, respectively, then performed a literature search using PubMed and ScienceDirect to identify genes within each sex-specific set that have been associated with air pollution-related health outcomes. Specifically, we searched for the name of each gene in combination with any of the following diseases or processes: allergy (Magnussen et al. 1993), chronic obstructive pulmonary disease (COPD) (Ko et al. 2007), asthma (Bowatte et al. 2015), lung cancer (Raaschou-Nielsen et al. 2011), cardiovascular disease (CVD) (Mills et al. 2009), cerebrovascular disease (CeVD) (Johnson et al. 2010), Alzheimer’s disease (Finkelstein and Jerrett 2007) and cognition (Dadvand et al. 2015). Genes with lowest *p*-values and proven link to air pollution (AP)-related diseases were chosen for validation. For men, *DNAJB5*, *RAC3*, *EAPP*, *HDLBP*, *PRG2*, *PER1*, *PIK3R1*, and *SLA2* were selected for validation, whereas for women the gene list for validation included *AKAP6, LIMK1, SIRT7, ARHPGAP4, ATG16L2, TPM3, 5-HTR1B*, and *PYGO2*.

### Validation of Candidate Biomarker Genes by qPCR

***qPCR.*** Total RNA was reverse transcribed into cDNA by means of the GoScript Reverse Transcription System (Promega, Madison, WI, USA) using a Veriti 96 well Thermal cycler (TC-5000, Techne, Burlington, NJ, USA). A maximum of 3 μg of total RNA was used as input, and we used the protocol with an equal amount of oligo(dT) and random hexamer primers according to the manufacturer’s instructions. cDNA was stored at –20°C until further measurements. A quantitative real-time polymerase chain reaction (qPCR) was set up by adding 2 μL of a 10 ng/μL dilution of cDNA together with TaqMan Fast Advanced Master Mix (Life Technologies, Foster City, CA, USA) and PrimeTime^TM^ assay (Integrated DNA Technologies, Coralville, IA, USA), in a final reaction volume of 10 μL. Standard cycling conditions were used to analyze samples in a 7900HT Fast Real-Time PCR system (Life Technologies, Foster City, CA, USA). Expression of eight candidate biomarker genes for each sex was studied and Cq values were collected with SDS 2.3 software. Minimum Information on qPCR Experiments (MIQE) guidelines were taken into account (Bustin et al. 2009). Amplification efficiencies were between 90% and 110% for all assays. Raw data were processed to normalized relative gene expression values with qBase plus (Biogazelle, Zwijnaarde, Belgium) (Hellemans et al. 2007). Triplicates were run for all samples; technical replicates were included when the difference in Cq value was < 0.5. A set of three genes was used for data normalization, namely Hypoxanthine Phosphoribosyltransferase 1 (*HPRT*), Importine 8 (*IPO8*) and tyrosine 3-monooxygenase/tryptophan 5-monooxygenase activation protein, zeta (*YWHAZ*).

***Data analysis.*** Statistical analyses were carried out using SAS software (version 9.3, SAS Institute Inc., Cary, NC, USA). Continuous data were presented as mean ± standard deviation (SD) and categorical data as percentages (%) and frequencies. Models were adjusted for age, body mass index (BMI), SES, smoking (categorized as smokers, former smokers and never smokers), white blood cell counts (absolute number of leukocytes and percentage of neutrophils), time of day (< 1200 hours, 1200–1500 hours, 1500–1800 hours, > 2000 hours) and season (October–March or April–September) of blood sampling. *p*-Values < 0.05 were considered statistically significant, *p*-values corrected for multiple testing referred to as *q*-values. We plotted residuals for each gene to check whether significance was driven by outliers, these were removed where appropriate. To indicate significance of selected biomarker genes for each sex, we included an interaction term for sex in our main analysis. *p*-Values for the interaction term sex were calculated for all genes under study, not only those that were significant.

In validation analysis, we examined the association between gene expression and PM_10_ exposure, stratified by sex using linear regression models for the eight selected genes for each sex.

### ROC Curves Exposure Prediction

We calculated the ability to predict PM_10_ exposure based on expression of the set of eight validated genes significantly associated with PM_10_ exposure in the discovery cohort for each sex. For this purpose, we estimated sensitivity and specificity of the prediction using receiver operating characteristic (ROC) plots. Subjects were stratified according to their long-term PM_10_ exposure levels with the 75th percentile as cut-off point (25.7 μg/m^3^ annual mean for women, 24.5 μg/m^3^ for men). All analyses were repeated similarly using long-term PM_2.5_ exposure levels, the cut-off point for long-term PM_2.5_ exposure, or the 75th percentile of exposure was 16.0 μg/m^3^ for both men and women.

## Results

### Population Characteristics

Table 1 lists the characteristics of the study cohorts. All participants were of European origin. Distribution of sex, SES, age and BMI as well as exposure did not differ between the discovery and validation cohort. Both cohorts included just less than 50% men and age averaged (SD) 57.9 (4.3) years. Season of sampling differed between both cohorts, with sampling for the discovery phase of the study mainly occurring throughout the warm months of the year, whereas sampling for the validation study was mainly performed during the cold season. However, since we are working with average annual exposures over a 2-year period, this approach in itself corrects for the differences across seasons. Blood sampling was done ≤ 1500 hours for all discovery cohort participants, while most validation cohort participants had samples drawn after 1500 hours. The discovery cohort consistent only of non-smokers, whereas the validation cohort included smokers (*n* = 21).

**Table 1:** Study population and exposure characteristics.

Characteristics	Discovery cohort Men (*n* = 48)	Validation cohort Men (*n* = 75)	Discovery cohort Women (*n* = 50)	Validation cohort Women (*n* = 94)
Age, years	58.0 ± 4.5	58.0 ± 4.1	57.8 ± 4.2	58.1 ± 4.0
Body mass index, kg/m^2^	27.4 ± 3.5	26.1 ± 3.8	25.8 ± 3.7	25.5 ± 4.7
Socioeconomic status
Low	20 (41.7)	14 (18.7)	28 (56.0)	23 (24.5)
Medium	15 (31.3)	26 (34.7)	7 (14.0)	16 (17.0)
High	13 (27.1)	35 (46.7)	15 (30.0)	55 (58.5)
Smoking status
Nonsmokers	48 (100.0)	25 (33.3)	50 (100.0)	49 (52.1)
Former smoker	NA	43 (57.3)	NA	31 (33)
Current smoker	NA	7 (9.3)	NA	14 (14.9)
Season of blood sampling
Cold (October–March)	40 (83.3)	27 (36.0)	40 (80.0)	40 (42.6))
Warm (April–September)	8 (16.7)	48 (64.0)	10 (20.0)	54 (57.4))
Time of blood sampling
< 1200 hours	41 (85.4)	0 (0.0)	44 (88.0)	7 (7.5)
1200–1500 hours	7 (14.6)	20 (26.7)	6 (12.0)	25 (26.6)
1500–1800 hours	0 (0.0)	32 (42.7)	0 (0.0)	43 (45.7)
> 2000 hours	0 (0.0)	23 (30.7)	0 (0.0)	19 (20.2)
White blood cell count
Leukocytes (#/μL)	—	6981.5 ± 1632.1	—	6981.5 ± 1632.1
Neutrophils (%)	—	56.8 ± 8.1	—	56.8 ± 8.1
Exposure (μg/m^3^)
PM_10_ long-term	25.8 (21.5–30.4)	23.1 (20.3–27.4)	26.0 (20.5–35.3)	24.2 (20.4–28.2)
PM_2.5_ long-term	17.7 (15.5–20.8)	15.5 (14.5–17.6)	17.8 (15.4–20.9)	16.0 (14.7–18.3)
Note: Data are mean ± SE or number (%), exposure data are mean (5–95th percentile). —, data not available; NA, not applicable.				

### Gene Level Analysis

**Figure 2:**
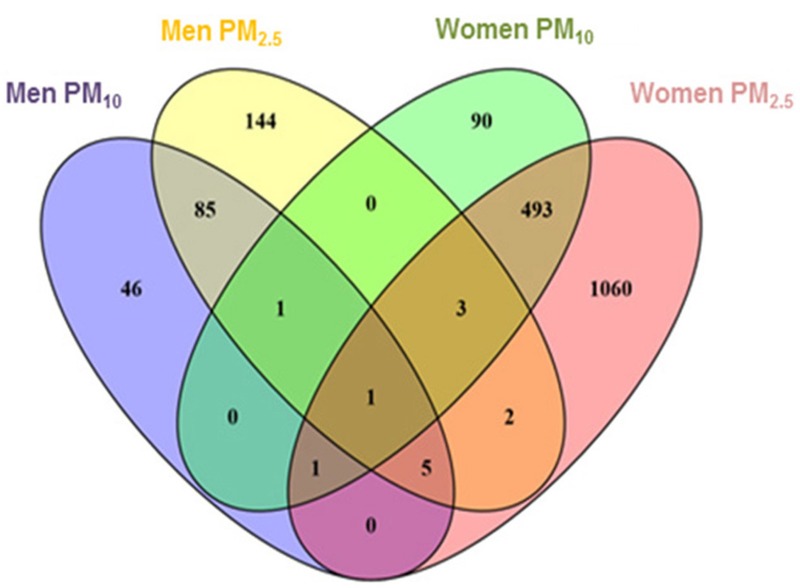
Venn diagram showing the overlap of all genes significantly associated with long-term PM_10_ and PM_2.5_ exposure in men and women in the discovery cohort.

Table 2 displays the 20 top genes for PM_10_ and PM_2.5_ exposure in men and women. Excel File Tables S1–S4 display the extended top 50 lists for each exposure/sex combination. An overview on the total number of significant genes identified in our analysis, indicating the overlap between men and women, is given in Figure 2. For 199 gene transcripts we noticed significant sex by particulate matter exposure (PM_10_) interactions (data not shown). The corresponding number of gene transcripts for PM_2.5_ with a significant sex by exposure interaction was 601 (data not shown). In men, there were significant associations between 47 genes and PM_10_ only, 149 genes and PM_2.5_ only, and there were 92 genes associated with both exposures. In women there were significant associations between 91 genes and PM_10_ only, 1,067 genes and PM_2.5_ only, and there were 498 genes associated with both exposures. We identified two genes in common between long-term PM_10_ exposure in men and women, namely *RAC3* and *DNAJB5*, respectively ranked as the 290th and 331st most significant genes with PM_10_ exposure in women (out of 592 genes). Furthermore *RAC3* was also significantly associated with long-term PM_2.5_ exposure in men and *DNAJB5* with long-term PM_2.5_ exposure in women. We did not observe any significant FDR-corrected *q*-values in the discovery phase of our study.

**Table 2:** Top 20 significant genes in association with 5-μg/m^3^ increase in long-term PM_10_ and PM_2.5_ exposure for men and women.

Rank no. gene	Men				Women			
	PM_10_		PM_2.5_		PM_10_		PM_2.5_	
	Gene	FC (95% CI)	Gene	FC (95% CI)	Gene	FC (95% CI)	Gene	FC (95% CI)
*1*	*EAPP*	1.15 (1.07, 1.24)	*ISL2*	2.45 (1.58, 3.78)	*ATG16L2*	0.81 (0.73, 0.90)	*EFNB1*	0.64 (0.53, 0.77)
*2*	*DCTN6*	1.23 (1.10, 1.38)	*HDLBP*	1.31 (1.14, 1.50)	*EFNB1*	0.79 (0.69, 0.89)	*SLC6A7*	1.52 (1.25, 1.86)
*3*	*DNAJB5*	1.36 (1.14, 1.63)	*B3GNT3*	1.42 (1.18, 1.70)	*SYTL1*	0.86 (0.79, 0.93)	*FXN*	0.73 (0.62, 0.85)
*4*	*ISL2*	1.55 (1.17, 2.06)	*RNF144*	1.83 (1.28, 2.62)	*SMG5*	0.84 (0.76, 0.92)	*SFPQ*	1.41 (1.18, 1.67)
*5*	*KIAA1914*	1.23 (1.07, 1.42)	*ATOH8*	2.24 (1.37, 3.66)	*TBC1D10C*	0.85 (0.78, 0.93)	*NACAL*	0.69 (0.58, 0.84)
*6*	*HDLBP*	1.14 (1.04, 1.24)	*RAC3*	1.62 (1.21, 2.18)	*NACAL*	0.81 (0.72, 0.91)	*ATG16L2*	0.72 (0.61, 0.86)
*7*	*B3GNT3*	1.19 (1.06, 1.34)	*ADCK1*	1.49 (1.17, 1.91)	*NFKBIE*	0.85 (0.78, 0.93)	*SLC24A2*	1.73 (1.28, 2.32)
*8*	*ATOH8*	1.55 (1.14, 2.10)	*DNAJB5*	1.62 (1.20, 2.18)	*CEMP1*	0.80 (0.70, 0.91)	*THEX1*	0.34 (0.19, 0.62)
*9*	*LSM12*	0.86 (0.77, 0.95)	*ALX3*	1.40 (1.13, 1.73)	*DCUN1D2*	0.84 (0.76, 0.93)	*TBC1D13*	0.78 (0.68, 0.90)
*10*	*ZNF187*	1.16 (1.04, 1.28)	*MAN2A2*	1.39 (1.13, 1.72)	*SLC6A7*	1.25 (1.10, 1.43)	*VAPB*	1.21 (1.09, 1.35)
*11*	*ARHGAP25*	1.11 (1.03, 1.20)	*DCTN6*	1.35 (1.11, 1.64)	*DHRSX*	1.21 (1.08, 1.36)	*TPM3*	0.44 (0.28, 0.70)
*12*	*SERF1B*	0.83 (0.72, 0.95)	*DAK*	1.34 (1.10, 1.64)	*TBC1D13*	0.86 (0.79, 0.94)	*CYB5D1*	0.39 (0.23, 0.67)
*13*	*ANXA1*	1.19 (1.05, 1.36)	*PER1*	1.37 (1.11, 1.69)	*SFPQ*	1.21 (1.08, 1.35)	*ZNF77*	0.61 (0.46, 0.81)
*14*	*TKTL1*	1.36 (1.09, 1.71)	*GUCA2B*	1.78 (1.20, 2.62)	*MAPK3*	1.19 (1.07, 1.32)	*GABRD*	0.40 (0.24, 0.67)
*15*	*PRG2*	1.29 (1.07, 1.56)	*ATXN7L3*	1.30 (1.09, 1.55)	*ZFYVE27*	0.91 (0.86, 0.96)	*NFKBIE*	0.78 (0.68, 0.90)
*16*	*PER1*	1.19 (1.05, 1.36)	*LSM12*	0.77 (0.64, 0.92)	*SLC39A2*	1.32 (1.11, 1.55)	*CEACAM3*	1.67 (1.24, 2.23)
*17*	*GUCA2B*	1.38 (1.09, 1.75)	*PRG2*	1.57 (1.15, 2.13)	*TSPAN4*	1.37 (1.13, 1.65)	*TSPAN4*	1.69 (1.25, 2.28)
*18*	*ST14*	1.20 (1.05, 1.37)	*ABL2*	1.40 (1.11, 1.78)	*DNAJC5*	0.87 (0.81, 0.95)	*GPR137*	0.64 (0.50, 0.83)
*19*	*CDV3*	0.84 (0.73, 0.96)	*MAST3*	1.27 (1.07, 1.49)	*MIA*	0.82 (0.73, 0.93)	*DNAJC5*	0.80 (0.70, 0.91)
*20*	*TTC30B*	1.20 (1.04, 1.37)	*PIK3R1*	1.46 (1.12, 1.89)	*CES2*	1.16 (1.06, 1.28)	*HSF1*	0.85 (0.77, 0.93)
Note: Rank no. gene indicates its hierarchy for that particular exposure and sex based on level of significance of the identified association, so gene ranked as no. 1 has the lowest *p*-value. FC, fold change.								

### Pathway Analysis

There were 1,251 and 966 pathways significantly associated with PM_10_ and PM_2.5_, respectively, in men, and 280 and 182 pathways significantly associated with PM_10_ and PM_2.5_ in women, based on uncorrected *p*-values. The top 5 identified pathways for each indicator of exposure are summarized in Table 3.

**Table 3:** The top five significant pathways defined by gene set enrichment analysis for each indicator of exposure.

Exposure/pathway	*q*‑Value	# measured/# genes in pathway
Men
PM_10_
Response to elevated platelet cytosolic Ca2+	3·11E-07	76/87
Prolactin signaling pathway	5·78E-07	61/72
Platelet degranulation	5·90E-07	71/82
Leukocyte transendothelial migration	1·25E-06	98/118
Signaling by insulin receptor	5‑18E‑06	89/109
PM_2.5_
Cell-cell communication	1·35E-08	95/130
Chagas disease (American trypanosomiasis)	1·40E-06	92/104
Signaling by type 1 insulin-like growth factor 1 receptor (IGF1R)	1·40E-06	76/96
Signaling by insulin receptor	1·93E-06	96/120
Insulin receptor signaling cascade	2·33E-06	74/76
Women
PM_10_
Respiratory electron transport, ATP synthesis by chemiosmotic coupling, and heat production by uncoupling proteins	2·08E-04	89/97
Packaging of telomere ends	3·98E-04	46/53
Electron transport chain	8·11E-04	94/103
Respiratory electron transport	9·59E-04	71/76
Telomere maintenance	1·50E-03	72/81
PM_2.5_
Respiratory electron transport	9·07E-04	81/92
Respiratory electron transport, ATP synthesis by chemiosmotic coupling, and heat production by uncoupling proteins	1·77E-03	99/113
Packaging of telomere ends	4·54E-03	45/52
Proteasome	4·93E-03	41/44
Transcriptional regulation by small RNAs	4·93E-03	95/106
Note: Pathways were identified using the Gene Set Enrichment Analysis Tool from the online Consensus Pathway Data Base (http://cpdb.molgen.mpg.de/). #, number.		

Long-term PM_10_ exposure in men is associated with response to elevated platelet cytosolic Ca^2+^, the prolactin signaling pathway and platelet degranulation. The 5th top significant pathway in association with PM_10_ exposure in men is signaling by insulin receptor, which ranks 4th when analyzing long-term PM_2.5_ exposure. Other pathways associated with PM_2.5_ exposure in men are cell-cell communication and signaling by Type 1 Insulin-like Growth Factor and Insulin receptor signaling cascade. For women, long-term PM_10_ exposure was associated with, in descending order of significance, respiratory electron transport, packaging of telomere ends, electron transport chain, respiratory electron transport and telomere maintenance. PM_2.5_ exposure was associated with respiratory electron transport, and the proteasome in women (Table 3).

### Transcriptome Signature in Relation to Long-Term Exposure

We selected eight genes that were significantly (*p* < 0.05) associated with long-term PM_10_ exposure in the microarray study and have a published link with air pollution-related disease (Table 4) for validation in an independent cohort. Of these we could confirm (i.e., they were also significantly associated with PM_10_ in the validation cohort based on uncorrected *p*-values, and associations were in the same direction as in the discovery cohort) two out of eight genes for men [DnaJ homolog, subfamily B, member 5 (*DNAJB5*), and E2F associated phosphoprotein (*EAPP*)] and one out of eight genes for women to be [Rho GTPase Activating protein 4 (*ARHGAP4*)] borderline significantly (*p* = 0.0535) associated with PM_10_ exposure (Table 4). *AKAP6* (*p* = 0.02) and *LIMK1* (*p* = 0.006) were significantly associated with PM_10_ in women in the validation cohort, albeit with significantly lower expression instead of higher expression as in the discovery cohort. We also tested the same sets of eight genes for each sex for associations with PM_2.5_ exposure in the validation cohort, since all but one of the candidate genes (*PYG02* in women, which also was not significant for PM_10_ in the discovery cohort) were significantly associated with long-term PM_2.5_ exposure in the discovery cohort. For PM_2.5_ exposure, we could confirm two out of eight genes [*DNAJB5* (borderline significant, *p* = 0.059) and *EAPP*] for men and four out of eight genes for women [*ARHGAP4*, *PYGO2*, sirtuin 7 (*SIRT7*) and Autophagy related 16-like 2 (*ATG16L2*)] (see Table S1). Excluding 21 current smokers (14 of 94 women and 7 of 75 men) from the validation cohort did not alter our conclusions, based on the similarity in the effect estimates, apart from expression of *ARHGAP4* in association with long-term PM_10_ exposure (see Table S2).

**Table 4:** Selection of biomarker candidate genes and their fold changes for an increase of 5 μg/m^3^ long-term PM_10_ exposure.

Gene name	Gene description	Gene function	Link to disease	Discovery cohort FC (95% CI)	*p*-Value	Validation cohort FC (95% CI)	*p*-Value	*q*-Value
Men
*DNAJB5*	DnaJ (Hsp40) homolog, subfamily B, member 5	Heat shock protein 40	CVD (Ago et al. 2008)	1.36 (1.14, 1.63)	0.0014	1.64 (1.20, 2.23)	0.0026	0.02
*RAC3*	Ras-related C3 botulinum toxin substrate 3 (rho family, small GTP binding protein Rac3)	Regulation of cellular responses (cell growth)	Lung cancer (Liu et al. 2015)	1.25 (1.04, 1.51)	0.024	1.26 (0.94, 1.96)	0.10	0.18
*EAPP*	E2F associated phosphoprotein	Cell cycle/apoptosis	Lung cancer (DeMuth et al. 1998)	1.15 (1.0, 1.24)	0.00055	1.18 (1.02, 1.38)	0.028	0.12
*HDLBP*	High density lipoprotein binding protein (vigilin)	Sterol metabolism	CVD (Husten 1998)	1.14 (1.04, 1.24)	0.0065	1.02 (0.88, 1.19)	0.75	0.86
*PRG2*	Proteoglycan 2	Eosinophil major basic protein	CVD (Melchior et al. 2013), asthma (Li et al. 2006)	1.29 (1.07, 1.56)	0.012	1. 29 (0.98, 1.71)	0.066	0.18
*PER1*	Period homolog 1 (Drosophila)	Circadian rhythm	CVD (Young et al. 2001)	1.19 (1.05, 1.36)	0.012	0.95 (0.74, 1.23)	0.72	0.86
*PIK3R1*	Phosphoinositide-3-kinase, regulatory subunit 1 (p85 alpha)	Insulin metabolism	Lung cancer (Lu et al. 2006)	1.22 (1.03, 1.43)	0.023	1.01 (0.82, 1.26)	0.91	0.91
*SLA2*	Src-like adaptor 2	SLAP adapter protein	CVD (Cherpokova et al. 2015)	1.22 (1.03, 1.44)	0.027	1.16 (0.97, 1.39)	0.11	0.18
Women
*AKAP6*	A kinase (PRKA) anchor protein 6	Regulatory subunit of protein kinase A	CVD (Oti et al. 2006)	1.21 (1.07, 1.36)	0.0036	0.72 (0.55–0.94)	0.017	0.05
*LIMK1*	LIM domain kinase 1	Regulation of actin filament dynamics	Lung cancer (Chen et al. 2013), Alzheimer’s (Heredia et al. 2006)	1.28 (1.06, 1.55)	0.01	0.75 (0.61–0.91)	0.0057	0.03
*SIRT7*	Sirtuin (silent mating type information regulation 2 homolog) 7 (S. cerevisiae)	Transcription repressor	CVD (Vakhrusheva et al. 2008)	0.89 (0.82, 0.96)	0.0038	0.80 (0.6–1.07)	0.14	0.22
*ARHGAP4*	Rho GTPase Activating protein 4	Regulation of small GTP-binding proteins from the RAS superfamily	Cognition (Huang et al. 2012)	0.88 (0.81, 0.95)	0.0035	0.62 (0.38–1.00)	0.054	0.11
*ATG16L2*	Autophagy related 16-like 2 (S. cerevisiae)	Autophagy	CVD (Magné et al. 2015)	0.81 (0.73, 0.90)	0.00028	0.81 (0.59–1.11)	0.19	0.25
*TPM3*	Tropomyosin 3	Actin-binding protein	Lung cancer (Rostila et al. 2012)	0.65 (0.48, 0.88)	0.0086	1.02 (0.83–1.26)	0.85	0.85
*5-HTR1B*	5-Hydroxytryptamine (serotonin) receptor 1B	Neurotransmitter/ vasoconstriction	CVD (Iwabayashi et al. 2012)	1.31 (1.08, 1.59)	0.0097	1.28 (0.49–3.34)	0.62	0.71
*PYGO2*	Pygophus homolog 2	Related to Wnt signaling	Lung cancer (Liu et al. 2013)	0.93 (0.85, 1.01)	0.097	0.75 (0.61–0.92)	0.0078	0.03
Note: Models adjusted for age, BMI, SES, smoking (validation cohort), leukocyte and neutrophil count, daytime of blood sampling and season. *p*-Values corrected for multiple testing are represented as *q*-values.								

### Validation Set

**Figure 3:**
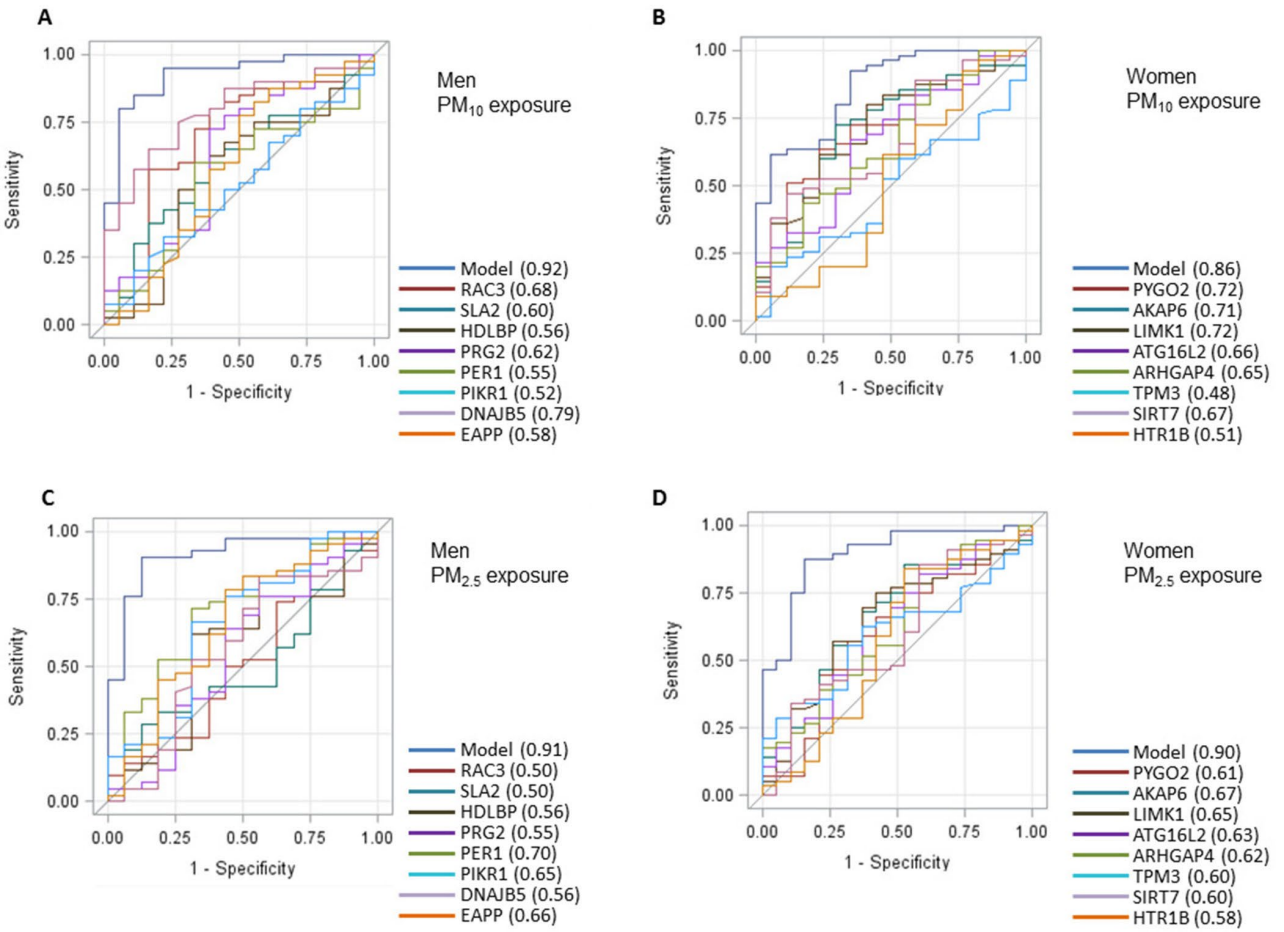
Receiver operating characteristics (ROC) curve for leukocyte gene expression of gene sets distinguishing between high and low long-term PM_10_ or PM_2.5_ exposure, respectively, based on the eight genes selected for validation for each sex. (*A*) performance of gene set consisting of *DNAJB5*, *RAC3*, *SLA2*,* HDLBP*,* PRG2*,* PER1*,* PIK3R1*, and *EAPP* to dinstinguish between high and low PM_10_ exposure in men (above 75th percentile corresponding to 24.5 μg/m^3^) and low (< 24.5 μg/m^3^) and (*B*) performance of gene set consisting of *ARHGAP4*, *AKAP6, PYGO2, HTR1B, ATG16L2, SIRT7*,* TPM3* and *LIMK1* in women to distinguish between high (above 75th percentile corresponding to: 25.7 μg/m^3^) and low (< 25.7 μg/m^3^) long-term residential PM_10_ exposure. (*C*) Performance of same male-specific gene set in men and (*D*) female-specific gene set in women to distinguish between high (above 75th percentile corresponding to: 16.0 μg/m^3^) and low (< 16.0 μg/m^3^) long-term residential PM_2.5_ exposure.

To determine whether gene expression candidate biomarkers identified in the discovery cohort were robust exposure markers, we performed ROC curve analysis with long-term PM_10_ exposure level 24.5 μg/m^3^ (75th percentile) as cut-off point in men. Figure 3A shows the sensitivity and 1 minus specificity (false positive ratio) of PM_10_ exposure levels for men in association with the candidate biomarker genes. The model including the eight genes in men had an area under the curve (AUC) value of 0.92 [95% confidence interval (CI): 0.85, 1.00; *p* = 0.0002]. In women the model including the eight genes had an AUC of 0.86 (95% CI: 0.76, 0.96; *p* = 0.004) (Figure 3B, cut-off point 25.7 μg/m^3^). The combined gene set perfomed better both in men and women than the individual genes. Similarly, for PM_2.5_ exposure prediction, the model for men had an AUC of 0.91 (95% CI: 0.83, 0.97; *p* = 0.007) (Figure 3C), the model for women had an AUC of 0.90 (95% CI: 0.81, 0.98; *p* = 0.0002) (Figure 3D).

## Discussion

We identified and validated transcriptome signatures that are associated with long-term exposure to particulate air pollution in apparently healthy men and women. These sets of eight sex-specific genes were predictive of exposure in the validation cohort, and including all eight genes in one model provided a better prediction than the eight genes individually. We found *DNAJB5* and *EAPP* in men and *ARHGAP4* in women based on a discovery set and a validation analysis to be significantly associated with PM_10_ exposure. Besides *ARHGAP4*, our PM_2.5_-exposure analysis for women identified *PYGO2*, *SIRT7,* and *ATG16L2* as significantly associated with particulate matter exposure. However, we cannot assume these associations indicate causal relations due to the observational nature of our study. ROC analysis revealed excellent separation between individuals with high and low exposure to long-term particulate air pollution using the genes selected for validation. We believe gene expression levels have the potential to be used as biomarkers of exposure and effect with high specificity to link particulate air pollution to its health consequences, as these can be measured at the personal level rather than be obtained through exposure modelling at the population level. Further studies looking at different age and ethnic groups are warranted to explore the capabilities of gene expression levels as predictors in more depth. Longitudinal studies that monitor disease incidence, exposure and gene expression over time would be excellent to provide more insights.

We observed different transcriptomic expression levels in association with particulate air pollution exposure in men and women. Sex-specific differences may be explained by differences in inflammatory responses between men and women. Immunologic differences between men and women have been reported based on gene expression profiles in blood between smokers and nonsmokers, where women seem to have a more specific (involving less extensive pathways) immunologic response to smoking than men (Faner et al. 2014). Furthermore, sex-specific associations were also reported for microarray expression profiles in relation to environmental exposure to diverse compounds such as PCBs, dioxin, benzene, and PAHs (De Coster et al. 2013). The sex-specific associations between PM and gene expression that we observed are in line with previous reports of sex-specific associations with other exposures. As such, prenatal exposure to bisphenol A (BPA) led to differential responses in murine placentae of female and male embryos (Imanishi et al. 2003). Prenatal stress exposure in rats was associated with sex-specific differences in gene expression and behavioral effects in male and female offspring (Van den Hove et al. 2013). This study clearly shows the same biological exposure (i.e., prenatal stress) leads to a highly differential response in male and female offspring.

To date, limited human data is available on microarray gene expression profiling in response to air pollution exposure. However, in an attempt to study the effects of *in utero* carcinogenic exposures, gene expression profiles in cord blood from 111 babies participating in the Norwegian BraMat cohort were assessed and correlation analyses of gene expression levels with biomarkers of exposure measured showed variable numbers of significantly correlating genes. Overall, separate analyses for male and female newborns resulted in higher numbers of significantly correlating genes per sex with low overlap of similarly expressed genes between the two sexes, thus indicating a clear sex-specific toxicogenomic response. More specifically, the authors reported only 1 gene in common between girls (39 significant genes) and boys (331 significant genes) for dioxin exposure (Hochstenbach et al. 2012).

Given evidence of the differential responses to PM exposure both at the gene and pathway levels between men and women, we hypothesize that different pathways could lead to the same disease outcome in both sexes. Recently, it was reported that the same personal exposure (i.e., smoking) could lead to disease in a differential manner in men and women. As such, Paul and Amundson (2014) described microarray analysis in smokers and nonsmoking men and women. They utilized a population of 24 middle-aged smoking men (*n* = 12) and women (*n* = 12) and an equal number of nonsmoking controls. The gene set correlated with smoking in men was incapable of separating female smokers from nonsmokers and vice versa. They identified a large number of oncogenic pathway gene sets that were significantly different in female smokers compared with male smokers with Gene Set Enrichment Analysis of microarray data. In addition, functional annotation with Ingenuity Pathway Analysis (IPA) identified smoking-correlated genes associated with biological functions in male and female smokers that are directly relevant to well-known smoking related pathologies. However, these relevant biological functions were overrepresented in female smokers compared with male smokers. Identified pathway categories in women were xenobiotic metabolism signaling, actin metabolism signaling, clathrin-mediated signaling, eicosanoid signaling, thrombin signaling, tight junction signaling, molecular mechanism of cancer, and natural killer cell signaling (Paul and Amundson 2014).

The expression of *ARHGAP4* was borderline significantly associated with long-term PM_10_ exposure in women in the discovery cohort, and borderline significant (*p* = 0.0535) in the validation cohort. *ARHGAP4, SIRT7*, and *ATG16L2* were furthermore significantly associated with long-term PM_2.5_ exposure in women in the discovery cohort and validation cohort.

*ARHGAP4* is a RhoGAP that regulates the cytoskeletal dynamics that control cell motility and axon outgrowth (Vogt et al. 2007). Pygosus 2 (*PYGO2*) is a component of the Wnt signaling pathway required for β-catenin/T-cell factor (TCF)-dependent transcription and has been shown to be upregulated in lung cancer both *in vitro* in non-small cell lung cancer cell lines and *in vivo* in human primary tumor tissue samples (Zhou et al. 2014).

*In vitro* experiments using hematopoietic stem cells from sirtuin 7 (*SIRT7*) knockout mice have shown *SIRT7* regulates mitochondrial activity and its inactivation causes reduced quiescence, increased mitochondrial protein folding stress, and compromised regenerative capacity of hematopoietic stem cells (Mohrin et al. 2015; Liu and Chen 2015). Mitochondrial DNA and function have been shown to be associated with chronic air pollution exposure in populations of newborns (Janssen et al. 2012) and elderly men (Zhong et al. 2016), hence NAD-dependent deacetylase SIRT7 might provide insight into a molecular mechanism underlying the mitochondrial damage following air pollution exposure. Autophagy related 16-like 2 (*ATG16L2*) is a core autophagy gene. Previously, we found in newborns epigenetic modifications in the mitochondrial genome, in association with PM_2.5_ exposure during gestation and placental mtDNA content, which could reflect signs of mitophagy and mitochondrial death (Janssen et al. 2012).

The expression of the genes *DNAJB5* and *EAPP* were significantly associated with PM_10_ air pollution exposure in men, in the discovery cohort, and in the validation cohort. DNAJB5 is a member of the evolutionarily conserved DNAJ/HSP40 family of proteins, which regulate molecular chaperone activity by stimulating ATPase activity (Ohtsuka and Hata 2000). DNAJB5 contains a cysteine-rich domain which renders the protein sensitive to ROS. The protein forms a multiprotein complex together with Trx1 and class II histone deacetylases (HDACs) that functions as a master negative regulator of cardiac hypertrophy (Ago et al. 2008). E2F-associated phospho-protein (EAPP) is a nuclear phosphoprotein that interacts with the activating members of the E2F transcription factor family. *In vitro* overexpression of EAPP increased the fraction of G1 cells and led to heightened resistance against DNA damage. EAPP itself becomes upregulated after DNA damage and stimulates the expression of p21 independently of p53 (Andorfer and Rotheneder 2011).

In pathway analyses, we identified several respiratory chain related pathways significantly associated with long-term PM_10_ and PM_2.5_ exposure in women. Rossner et al. (2015) reported deregulation of expression of respiratory chain, oxidative phosphorylation, and mitochondrial membrane pathways when comparing gene expression profiles in adult nonsmoking men from a heavily polluted area versus a control region in the Czech Republic across different seasons (winter and summer 2009 and winter 2010).

Although sex-related differences have been observed for different environmental pollutions, to our knowledge, this is the first study on microarray gene expression profiles in association with long-term air pollution exposure among middle-aged men and women.

Our study has strengths and limitations. We did our investigations in two independent cohorts for discovery and validation, using the same exposure modeling and the gold standard qPCR as validation tool (Canales et al. 2006). Although sample size for the discovery cohort was limited, we believe validation in an independent cohort based on a reliable method such as qPCR indicates the robustness of our analyses. Our study also has its limitations inherent to the cross-sectional nature of our study. We used 2010–2012 air pollution data to develop our high-resolution exposure models, which we applied to the participants’ baseline addresses (2004). Studies in the Netherlands (Brauer et al. 2003), Italy (Rome) (Rosenlund et al. 2008), the United Kingdom (Briggs et al. 2000), and Canada (Vancouver) (Henderson et al. 2007) have shown that during periods of about 10 years and longer, existing land use regression models predicted historic spatial contrasts well. The use of a relatively homogenous population limits the potential generalizability of our study to populations with different ages, races and ethnicities, or locations. Lastly, our study design did not allow to control for cell counts in the discovery phase of the study. As cell counts were not performed on the samples for microarray analysis, and there is no good means for imputation of these values for Agilent 4 × 44 K arrays during data analysis, we were not able to control for this.

This is the first time levels of gene expression of candidate genes have been used to accurately predict air pollution exposure levels (PM_10_, PM_2.5_). For this purpose, we have established ROC curves based on the genes selected for validation in an independent cohort and were able to separate low- (< 75th percentile) from high- (> 75th percentile) exposed individuals. ROC curves are commonly used to compare the diagnostic performance of two or more tests, as they give a good indication of both the sensitivity and specificity of the studied test (Greiner et al. 2000). As such, it has been demonstrated that gene expression signatures can predict survival for instance in pancreatic (Newhook et al. 2014) or non-small cell lung cancer (Lu et al. 2006). In 2009, this technique was applied for the first time in an environmental epidemiology setting, showing that specific DNA methylation patterns could accurately predict the relationship between exposure to airborne PAHs and childhood asthma incidence. Perera et al. (2009) investigated PAH levels in cord blood samples from 20 newborns and replicated the association between PAH levels and candidate region methylation in 56 other newborns from the Columbia Center for Children’s Environmental Health (CCCEH) cohort that recruits nonsmoking Dominican and African American women and their children residing in different areas of New York in the United States (Perera et al. 2009). However, the application of this approach to the field of gene expression data in association with air pollution exposure is novel.

In ROC curve analysis, an AUC of 0.80 is considered a ROC curve with good separation characteristics, and an AUC of 0.90 is considered excellent, in its ability to distinguish between true and false positives. We have identified sex-specific gene-sets that fulfill these criteria for PM_10_ and PM_2.5_ exposure. However, we must interpret the current set within the context of its limitations inherent to the cross-sectional nature of our study.

## Conclusions

In conclusion, microarray analysis has identified different gene-expression levels in response to long-term air pollution in men and women. From gene-level analysis, candidate biomarker genes with a reported link to AP-related disease were selected and validated (i.e., significantly associated with PM exposure with the same direction of regulation of expression) in an independent cohort. For men, we propose *DNAJB5* and *EAPP* as biomarkers of exposure. For women, we identified *ARHGAP4*, *PYGO2*, *SIRT7*, and *ATG16L2* as biomarker genes of exposure. ROC analysis revealed that the genes were able to predict high or low PM_10_ exposure accurately. Prospective studies in other populations are needed to confirm our findings with regard to sex-specific expression of these genes in association with PM exposure. Furthermore, it would be highly relevant to analyze the gene expression of these sex-specific gene-sets in cohorts with higher PM exposure as well as in subjects at different stages of life, including the more vulnerable stages such as early childhood and puberty.

## Supplemental Material

(231 KB) PDF

(59 KB) ZIP
