# Supplementary material for: Sex-Specific Associations between Particulate Matter Exposure and Gene Expression in Independent Discovery and Validation Cohorts of Middle-Aged Men and Women
Source: Environ Health Perspect. 2016 Oct 14;125(4):660–9. doi: 10.1289/EHP370 (PMC5381989; doi:10.1289/EHP370)
Supplement: (231 KB) PDF [file EHP370.s001.acco.pdf]

Environ Health Perspect
DOI: 10.1289/EHP370

Note to readers with disabilities: *EHP* strives to ensure that all journal content is accessible to all readers. However, some figures and Supplemental Material published in *EHP* articles may not conform to 508 standards due to the complexity of the information being presented. If you need assistance accessing journal content, please contact ehponline@niehs.nih.gov. Our staff will work with you to assess and meet your accessibility needs within 3 working days.

Supplemental Material

Sex-Specific Associations between Particulate Matter Exposure and Gene Expression in Independent Discovery and Validation Cohorts of Middle-Aged Men and Women

Karen Vrijens, Ellen Winckelmans, Maria Tsamou, Willy Baeyens, Patrick De Boever, Danyel Jennen, Theo M. de Kok, Elly Den Hond, Wouter Lefebvre, Michelle Plusquin, Hans Reynders, Greet Schoeters, Nicolas Van Larebeke, Charlotte Vanpoucke, Jos Kleinjans, and Tim S. Nawrot

Table of Contents

Table S1. Associations of the selected candidate biomarker genes with long term $PM_{2.5}$ exposure in the discovery and validation cohort.

Table S2. Associations between long term PM_{10} and $PM_{2.5}$ exposure and log2- transformed gene expression for the complete validation cohort and the validation cohort excluding current smokers.

Additional Files

- **Supplemental Code and Data ZIP File Index**
- **Supplemental Code and Data ZIP File**
  - **Excel File Tables S1-S4**
    - **Excel File Table S1.** The top 50 genes associated with a 5-µg/m^3 increase in PM_{10} in men, and p-values, q-values, and fold changes (with 95% CI) for each gene.
    - **Excel File Table S2.** The top 50 genes associated with a 5-µg/m^3 increase in $PM_{2.5}$ in men, and p-values, q-values, and fold changes (with 95% CI) for each gene.
    - **Excel File Table S3.** The top 50 genes associated with a 5-µg/m^3 increase in PM_{10} in women, and p-values, q-values, and fold changes (with 95% CI) for each gene.

Excel File Table S4. The top 50 genes associated with a 5-μg/m^3 increase in $PM_{2.5}$ in women, and p-values, q-values, and fold changes (with 95% CI) for each gene.

Table S1. Associations of the selected candidate biomarker genes with long term $PM_{2.5}$ exposure in the discovery and validation cohort

	Gene description	Gene function	Link to disease	Discovery cohort FC (95% CI)	p-value	Validation cohort FC (95% CI)	p-value	q-value
Men								
DNAJB5	DnaJ (Hsp40) homolog, subfamily B, member 5	Heat shock protein 40	CVD[40]	1.62 (1.20, 2,18)	0.003	2.16 (1.00, 4.73)	0.06	0.16
RAC3	ras-related C3 botulinum toxin substrate 3 (rho family, small GTP binding protein Rac3)	Regulation of cellular responses (cell growth)	Lung cancer [50]	1.62 (1.21, 2.18)	0.003	1.26 (0.94, 1.69)	0.32	0.62
EAPP	E2F associated phosphoprotein	Cell cycle/Apoptosis	Lung cancer [51]	1.31 (1.14, 1.50)	0.009	1.55 (1.08, 2.22)	0.02	0.16
HDLBP	high density lipoprotein binding protein (vigilin)	Sterol metabolism	CVD [52]	1.20 (1.06, 1.37)	0.0004	1.00 (0.69, 1.44)	0.77	0.77
PRG2	Proteoglycan 2	Eosinophil major basic protein	CVD [53], asthma [54]	1.57 (1.15, 2.13)	0.0069	1.30 (0.63, 2.71)	0.48	0.62
PER1	period homolog 1 (Drosophila)	Circadian rhythm	CVD [55]	1.37 (1.11, 1.69)	0.0061	0.52 (0.29, 0.96)	0.04	0.16
PIK3R1	phosphoinositide-3-kinase, regulatory subunit 1 (p85 alpha)	Insulin metabolism	Lung cancer [56]	1.45 (1.12, 1.89)	0.0079	1.25 (0.74, 2.10)	0.41	0.62
SLA2	Src-like adaptor 2	SLAP adapter protein	CVD [57]	1.44 (1.10, 1.90)	0.01	1.16 (0.73, 1.85)	0.54	0.62
Women								
AKAP6	A kinase (PRKA) anchor protein 6	Regulatory subunit of protein kinase A	CVD [58]	1.39 (1.26, 1.67)	0.002	0.44 (0.23, 0.81)	0.01	0.03
LIMK1	LIM domain kinase 1	Regulation of actin filament dynamics	Lung cancer [59] Alzheimer's [60]	1.51 (1.29, 2.04)	0.01	0.64 (0.41, 0.99)	0.05	0.07
SIRT7	sirtuin (silent mating type information regulation 2 homolog) 7 (S. cerevisiae)	Transcription repressor	CVD [61]	0.85 (0.80, 0.97)	0.02	0.44 (0.22, 0.86)	0.02	0.04
ARHGAP4	Rho GTPase Activating protein 4	regulation of small GTP-binding proteins from the	cognition [62]	0.82 (0.76, 0.93)	0.005	0.16 (0.05, 0.47)	0.002	0.008

		RAS superfamily						
ATG16L2	autophagy related 16-like 2 (S. cerevisiae)	Autophagy	CVD [63]	0.72 (0.66, 0.86)	0.0005	0.59 (0.37. 0.94)	0.03	0.05
TPM3	Tropomyosin 3	Actin-binding protein	Lung cancer [64]	0.44 (0.35, 0.70)	0.001	1.38 (0.85, 2.23)	0.20	0.20
5-HTR1B	5-hydroxytryptamine (serotonin) receptor 1B	Neurotransmitter/ vasoconstriction	CVD [65]	1.16 (1.38, 2.19)	0.004	5.35 (0.63, 45.5)	0.13	0.15
PYGO2	Pygophus homolog 2	Related to Wnt signaling	Lung cancer [66]	0.91 (0.85, 1.04)	0.17	0.48 (0.46, 0.51)	0.002	0.008

Models adjusted for age, BMI, SES, smoking (validation cohort), leukocyte and neutrophil count, daytime of blood sampling and season. FC= fold change calculated for an increase in $PM_{2.5}$ of 5 $\mu g/m^3$. Q-value= corrected p-value for multiple testing.

Table S2: Associations between long term PM_{10} and $PM_{2.5}$ exposure and log2- transformed gene expression for the complete validation cohort and the validation cohort excluding current smokers.

**PM_{10}**	Current smokers included		Current smokers excluded		**$PM_{2.5}$**	Current smokers included		Current smokers excluded	
Gene name	Estimated effect (95% CI)	p-value	Estimated effect (95% CI)	p-value		Estimated effect (95% CI)	p-value	Estimated effect (95% CI)	p-value
Men									
DNAJB5	1.64 (1.20, 2.23)	0.003	1.82 (1.30-2.56)	0.001		2.16 (1.00, 4.73)	0.06	2.96 (1.20, 7.29)	0.02
RAC3	1.26 (0.94, 1.96)	0.10	1.27 (0.92-1.76)	0.15		1.26 (0.94, 1.69)	0.32	1.50 (0.65, 3.43)	0.34
EAPP	1.18 (1.02, 1.38)	0.03	1.15 (0.98, 1.36)	0.09		1.55 (1.08, 2.22)	0.02	1.52 (1.02, 2.29)	0.05
HDLBP	1.02 (0.88, 1.19)	0.75	0.98 (0.84-1.15)	0.84		1.00 (0.69, 1.44)	0.77	0.94 (0.64, 1.39)	0.89
PRG2	1. 29 (0.98, 1.71)	0.07	1.09 (0.77-1.53)	0.64		1.30 (0.63, 2.71)	0.48	0.92 (0.32, 2.27)	0.85
PER1	0.95 (0.74, 1.23)	0.72	0.99 (0.74-1.33)	0.93		0.52 (0.29, 0.96)	0.04	1.94 (0.94, 4.05)	0.08
PIK3R1	1.01 (0.82, 1.26)	0.91	0.96 (0.75-1.22)	0.72		1.25 (0.74, 2.10)	0.41	1.19 (0.24, 2.62)	0.58
SLA2	1.16 (0.97, 1.39)	0.11	1.16 (0.95, 1.41)	0.16		1.16 (0.73, 1.85)	0.54	1.05 (0.61, 1.80)	0.87
Women									
AKAP6	0.72 (0.55-0.94)	0.017	0.74 (0.54-1.01)	0.06		0.44 (0.23, 0.81)	0.01	0.50 (0.26, 1.00)	0.05
LIMK1	0.75 (0.61-0.91)	0.0057	0.79 (0.61-1.02)	0.07		0.64 (0.41, 0.99)	0.05	0.77 (0.47, 1.27)	0.31
SIRT7	0.80 (0.6-1.07)	0.14	0.93 (0.66-1.32)	0.69		0.44 (0.22, 0.86)	0.02	0.61 (0.28, 1.33)	0.22
ARHGAP4	0.62 (0.38-1.00)	0.054	0.77 (0.43-1.37)	0.37		0.16 (0.05, 0.47)	0.002	0.25 (0.07, 0.85)	0.03
ATG16L2	0.81 (0.59-1.11)	0.19	0.89 (0.61-1.31)	0.70		0.59 (0.37. 0.94)	0.03	0.72 (0.43, 1.20)	0.21
TPM3	1.02 (0.83-1.26)	0.85	1.00 (0.78-1.30)	0.98		1.38 (0.85, 2.23)	0.20	1.37 (0.79, 2.39)	0.27
5-HTR1B	1.28 (0.49-3.34)	0.62	0.79 (0.25-2.53)	0.70		5.35 (0.63, 45.5)	0.13	2.54 (0.21, 30.4)	0.46

PYGO2	0.75 (0.61-0.92)	0.008	0.78 (0.60-1.01)	0.06	0.48 (0.46, 0.51)	0.002	0.58 (0.33, 1.01)	0.06
